# In Vitro Comparison of Mechanical and Esthetic Properties of Different Universal-Shade Resin Composites After Simulated Aging

**DOI:** 10.3390/ma19132778

**Published:** 2026-06-30

**Authors:** Md Sofiqul Islam, Smriti Aryal A C, Mohamed Ahmed Elsayed, Vivek Padmanabhan, Misbah Sultana, Nada Tawfig Hashim, Upoma Guha, Muhammed Mustahsen Rahman

**Affiliations:** 1 Department of Operative Dentistry, RAK College of Dental Sciences, RAK Medical and Health Sciences University, Ras Al-Khaimah P.O. Box 12973, United Arab Emirates; 2 Department of Oral and Craniofacial Health Sciences, College of Dental Medicine, University of Sharjah, Sharjah P.O. Box 27272, United Arab Emirates; saryalac@sharjah.ac.ae; 3 Department of Endodontics, RAK College of Dental Sciences, RAK Medical and Health Sciences University, Ras Al-Khaimah P.O. Box 12973, United Arab Emirates; mohamed.elsayed@rakmhsu.ac.ae; 4 Department of Endodontics, Faculty of Dentistry, Assiut University, Assiut 71516, Egypt; 5 Department of Pediatric Dentistry, RAK College of Dental Sciences, RAK Medical and Health Sciences University, Ras Al-Khaimah P.O. Box 12973, United Arab Emirates; vivek.padmanabhan@rakmhsu.ac.ae; 6 Research Center, RAK College of Dental Sciences, RAK Medical and Health Sciences University, Ras Al-Khaimah P.O. Box 12973, United Arab Emirates; misbah.sultana@rakcods.com; 7 Department of Periodontology, RAK College of Dental Sciences, RAK Medical and Health Sciences University, Ras Al-Khaimah P.O. Box 12973, United Arab Emirates; nada.tawfig@rakmhsu.ac.ae (N.T.H.); mustahsen@rakmhsu.ac.ae (M.M.R.); 8 Department of Adult Restorative Dentistry, College of Dentistry, University of Nebraska Medical Center, 4000 East Campus Loop South, Lincoln, NE 68583-0740, USA; uguha@unmc.edu

**Keywords:** universal-shade resin composite, micro-hybrid resin composite, hardness, elastic modulus, creep, solubility, color, gloss, thermocycling, durability

## Abstract

**Highlights:**

- MHRC showed higher hardness, modulus, and creep resistance than USRCs.
- Several USRCs demonstrated esthetic performance comparable to MHRC.
- Some USRCs exhibited superior color and gloss stability than MHRC.
- MHRC remains preferable when mechanical durability is critical.
- USRCs can simplify shade selection while maintaining esthetic outcomes.
- Material-specific selection is essential due to performance variability.

**Abstract:**

**Background**: Universal-shade resin composites (USRCs) have gained popularity for their simplified shade-matching and reduced chairside time. However, their long-term performance under oral environmental stresses remains uncertain. This study aimed to compare the mechanical and esthetic properties of five commercially available single-shade resin composites and one conventional micro-hybrid resin composite (MHRC) after simulated aging. **Methods**: A total of 18 disc-shaped specimens (10 mm circular × 3 mm thick) were prepared from five USRCs (Omnichroma, Zenchroma, A-Uno, TRANSCEND, and Ecosite One) and one MHRC (Clearfil AP-X). The polymerized specimen disk was polished using 2500 grit SIC paper. The mechanical properties, Vickers microhardness (HV), elastic modulus (EM) and creep (CR), and the esthetic properties, color and gloss, were measured after 24 h water storage and after 1-year stimulated aging. The data was analyzed using SPSS 27.0 software. A significance level of α = 0.05 was used for all statistical analyses. **Results**: Both the aging and types of material showed a significant effect on the mechanical and esthetic properties of the tested resin composite materials. The MHRC showed a significantly higher HV and EM compared to the USRC (*p* < 0.05). The MHRC showed significantly lower CR compared to USRC (*p* < 0.05). The color retention of USRCs was comparable to the MHRC (*p* > 0.05) and some USRCs showed superior gloss values compared with MHRC (*p* < 0.05). The solubility of USREs were comparable to MHRC (*p* > 0.05) except A-Uno which showed significantly higher solubility compared to MHRC (*p* < 0.05). **Conclusions**: Within the limitations of this in vitro study, it concludes that the USRC possesses inferior mechanical properties compared with the convention MHRC. Thus, their use should be limited when strong and durable mechanical properties are required under heavy occlusal stress. The use of USRCs could be beneficial for achieving a highly esthetic outcome of the restoration that does not includes the area that comes under heavy occlusal stress.

## 1. Introduction

Tooth-colored resin composite materials have become the restorative material of choice in contemporary esthetic dentistry, largely supplanting amalgam as the predominant direct restorative option worldwide. Their widespread adoption reflects a convergence of patient demand for esthetics, improved biocompatibility, adhesive bonding capabilities, and the ongoing refinement of composite formulations over several decades [1,2]. A conventional resin composite (RC) consists of an organic resin matrix that gets reinforced by inorganic filler particles, coupled with silane surface treatment [3]. The characteristics of filler-like content, particle size, and distribution critically determine the material’s mechanical performance, surface characteristics, and optical behavior.

RC materials are commonly classified based on the filler particle types and size, namely macro-fills, micro-fills, micro-hybrids, nano-fills, and nano-hybrids [4]. Among these, conventional micro-hybrid RCs featuring filler particle sizes between 0.4 and 1.0 µm have long represented the clinical benchmark for posterior restorations. This RC has demonstrated a good balance between mechanical strength and polish ability [5]. Clearfil AP-X by Kuraray Noritake Dental, Japan, is a widely studied micro-hybrid resin composite (MHRC) that has demonstrated reliable fracture toughness, microhardness, and dimensional stability across numerous in vitro investigations, making it a reliable comparator in studies evaluating novel RC systems [6,7].

Shade selection and matching is a significant clinical challenge associated with conventional RC restorations. Shade selection and matching using the classical VITA shade guide encompasses over 16 classical shades. To select an optimal RC shade, the clinicians must navigate metamerism, tooth translucency, value, chroma, and hue variations all under time pressure and variable operatory lighting conditions [8]. Color mismatch between the restoration and adjacent tooth structure remains one of the foremost reasons for esthetic dissatisfaction, failure and replacement of RC restoration [9].

To overcome the inaccurate shade selection and mismatch with surrounding tooth structure in clinical practice, a new generation of single-shade or universal-shade resin composites (USRCs) has emerged in the dental market over the past decade. These USRC materials are engineered to match a wide spectrum of tooth shades from a single paste, thereby simplifying the shade-matching procedure, reducing chairside time, and minimizing inventory demands [10]. The underlying optical mechanism of these USRC varies by product. Some of these USRCs employ spherical fillers that generate structural color through light interference, adapting to the surrounding tooth color [11]. Other USRCs utilize refractive index-matched filler and matrix systems or incorporate a Chromaviva technology designed to harmonize with diverse tooth shades [12,13]. Supporters of USRCs believe these materials can make cosmetic dentistry more accessible, easier to perform, and more consistent regardless of the patient or clinical environment [14]. Previous in vitro and clinical studies reported the blending potential and high perishability of these materials, which has made many dental professionals genuinely excited about their potential [15].

The long-term success of restorations relies on a combination of mechanical strength and esthetic qualities that must hold up over time in the challenging conditions of the mouth [16]. Mechanical property like Vicker’s micro-hardness is a measure of resistance to plastic deformation which directly correlates with resistance to occlusal wear [17]. Elastic modulus (EM) is another mechanical property that determines the stiffness of the restoration under functional loading and its ability to support the overlying enamel and distribute stress to the underlying dentin [18]. Creep (Cr), on the other hand, is time-dependent plastic deformation under sustained load, which is particularly relevant in posterior composites and can lead to clinically detectable cusp deflection and marginal degradation over time [19].

Like the mechanical viewpoint, from an esthetic perspective, surface gloss and color stability are the primary determinants of long-term restorative acceptability. The gloss of RC materials is governed by surface smoothness at the nanometer scale which is modulated by filler particle size, polishing protocol, and susceptibility to surface degradation [20]. The color stability of RC materials is quantified using the CIE L*a*b* color space, with color change (ΔE) calculated as the Euclidean distance between baseline and post-aging measurements. When ΔE exceeds beyond the acceptable thresholds, the color mismatch becomes clinically detectable or unacceptable to patients [21]. Mass stability of RC materials assessed through solubility and sorption testing reflects its susceptibility to aqueous degradation, which contributes to surface roughness, discoloration, and mechanical deterioration over time [22].

In vitro aging protocols are employed to simulate and accelerate the degradation that composite restorations experience over years of clinical service. Alternating the specimens between low (5 °C) and high (55 °C) temperature baths subjects materials to repeated thermal expansion and contraction stresses that replicate the cyclic thermal changes generated by dietary intake in the oral environment. Thermal stress promotes hydrolytic degradation of the silane coupling agent at the filler–matrix interface, plasticization of the polymer matrix by water sorption, and microcrack formation, collectively resulting in deterioration of both esthetic and mechanical properties [23].

The previous studies on single-shade composites mainly focused on evaluations of initial optical performance and short-term color matching efficacy like color blending with the surrounding tooth structure immediately after polymerization. A previous study demonstrated that Omnichroma achieved acceptable shade adaptation across a range of VITA shades under standardized illumination and confirmed its in vitro polish ability to be comparable to conventional nano-hybrid composites [24]. Other studies reported favorable initial optical properties for A-Uno when assessed against multiple tooth shades, and preliminary data for TRANSCEND and Ecosite One suggest comparable initial esthetic performance [25,26]. However, there is a lack of studies that have comprehensively and simultaneously evaluated the mechanical properties alongside esthetic properties and mass stability of multiple USRC after standardized thermal aging. This is a significant gap in the available evidence, especially considering how quickly these materials have been adopted in clinical practice. Without this information, dentists are left without a complete picture when deciding whether single-shade composites are truly suitable for long-term use particularly in posterior teeth, where chewing forces are greatest.

The aim of this study was to compare the mechanical and esthetic properties of five commercially available single-shade resin composites and one conventional micro-hybrid resin composite before and after simulated aging through thermocycling. The specific objectives of the study were as follows: (1) to measure mechanical properties of USRC by means of HV, EM and Cr before and after stimulated aging and to compare with that of conventional RC; (2) to measure esthetic properties of USRC by means of ΔE and GL before and after stimulated aging and to compare with that of conventional RC. The null hypothesis was that there would be no statistically significant difference in mechanical or esthetic properties among the tested resin composites following simulated aging (*p* > 0.05).

## 2. Materials and Methods

### 2.1. Study Design

This in vitro investigation received approval from the Research and Ethics Committee of Ras Al Khaimah Medical and Health Sciences University under approval number RAKMHSU/RES/In-Vitro/2025-26/16 before any experimental procedures were initiated.

### 2.2. Sample Size Calculation

G*Power statistical software (version 3.1.9.7) was used to calculate the required sample size for the present in vitro investigation. The estimation was performed through a priori power analysis using a repeated-measures ANOVA model to ensure adequate statistical power. The parameters used for the calculation were: effect size f = 0.5 (based on precious study by Islam MS et al., 2023 [16], confidence level of 95%, estimate power (1 − β) = 0.9, number of groups = 6, number of measurements = 2 and moderate correlation among repeated measures r = 0.5. This analysis indicated that a total of 18 specimens were required for the experiment.

### 2.3. Specimen Preparation

Five commercially available USRCs and one conventional MHRC were selected for this investigation. Material selection was based on current market availability, clinical relevance, and representativeness of distinct single-shade optical technologies. All materials were used strictly according to the manufacturers’ instructions and within their stated shelf-life dates. The composition and classification of the tested materials are summarized in Table 1. Disc-shaped specimens measuring 10 mm in diameter × 3 mm in thickness were fabricated for each material using a silicon mold. The thickness and the diameter of the specimen were determined to ensure its compatibility to be measured by all three devices and to eliminate background and surrounding artifact using color chromometer. The composite was condensed into the mold cavity in a single increment using a Teflon-coated plastic instrument, ensuring the absence of voids or air inclusions. A glass slide was pressed firmly against the mold to produce a flat, smooth surface, displacing excess material and standardizing specimen thickness. The specimens were then polymerized using a light cure (Eighteeth Curing Pen, Changzhou Sifary Medical Technology Co., Ltd., Changzhou City, Jiangsu, China) with a light output of 1500 mW/cm^2^. Each specimen was light-cured for 10 s per exposure, with 4 exposures in total. The light guide tip (curing width of about 10 mm), was positioned centrally perpendicular to the specimen surface at 0 mm distance, and a second 40 s exposure was applied to the opposing face. The specimens were then polished with 1000–2500 grit silicon carbide paper in a grinder–polisher (EcoMet 30, Buehler Ltd., Lake Bluff, IL, USA) to obtain a smooth surface.

### 2.4. Mechanical Properties Test

The specimens were the allocated for mechanical properties testing using a dynamic ultra-micro hardness tester (DUH-211S, Shimadzu Corporation, Kyoto, Japan). A nano size indenter with 50 mN force was applied on the specimen surface using a load–unload model. A total of 10 indentations were made on each specimen. The HV, EM and CR value were measured from each indentation. After that, the specimens were allocated for an artificial aging using an artificial aging device (Thermocycler THE-1100, SDMECHA-TRONIK GMBH, Feldkirchen-Westerham, Munich, Germany). To complete one cycle, the specimens were subjected to a hot thermal simulation at 55 °C and a cold thermal simulation at 50 °C in a water-submerged condition for 30 s each with a dwell time of 5 s. In total, 10,000 cycles were conducted to age the specimens by 1 year. After that, the mechanical properties tests using an ultra-hardness testing device were repeated to measure the HV, EM and CR value of 1-year aged specimens.

### 2.5. Esthetic Properties Test

Baseline color parameters (CIE L*, a*, b*) were recorded using a calibrated clinical spectrophotometer (VITA Easyshade V, VITA Zahnfabrik, KG, Bad Säckingen, Germany) positioned at the center of each specimen disc perpendicular to the surface. Three readings were taken per specimen and averaged. The instrument was calibrated against the manufacturer-supplied white ceramic reference tile before each measurement session. Color difference (ΔE) between baseline and post-aging measurements was calculated using both the conventional ΔE*ab formula.

$$\Delta E_{ab}=\sqrt{{\left(\Delta L^{*}\right)}^{2}+{\left(\Delta a^{*}\right)}^{2}+{\left(\Delta b^{*}\right)}^{2}}$$

Surface gloss was measured using a digital glossmeter (Novo-Curve 4 Glossmeter, Rhopoint Instruments, St Leonards-on-Sea, East Sussex, UK) at a 60° geometry. Five measurements were taken at different locations on the polished surface of each specimen and the mean was recorded in gloss units (GU). The glossmeter was calibrated against a standard black glass tile (GU = 96.2) before each session. The readings were repeated after 10,000 cycles of thermocycling.

### 2.6. Mass Stability Testing

The initial weight (W1) of each disc was recorded using an analytical balance (BA-E1004, Infitek Co., Ltd., Shanghai, China). The specimens were then allocated for 10,000 cycles of thermocycling. The readings were repeated after the thermocycling (W2). The solubility SL was calculated using the following formula: SL (/gm) = (W1 − W2)/W1.

### 2.7. Statistical Analysis

Statistical analyses were performed using IBM SPSS Statistics version 27.0 (IBM Corp., Armonk, NY, USA). Descriptive statistics were calculated to summarize the collected data and assess data distribution patterns. The normality of the variables was examined using the Shapiro–Wilk test. As the data met the assumptions of normality, parametric statistical methods were applied. Repeated-measures analysis of variance (ANOVA) was used to investigate the influence of aging on the mechanical, physical, and esthetic properties of the tested materials. Pairwise comparisons among groups were subsequently conducted using Tukey’s post hoc test. For color difference (ΔE) values that did not satisfy parametric assumptions, the non-parametric Kruskal–Wallis test was employed to compare differences among the study groups. A significance level of *p* < 0.05 was adopted for all statistical analyses. A significance level of α = 0.05 was used for all statistical analyses, and differences were considered statistically significant when *p* < 0.05. The graphs were generated in GraphPad Prism 10.2.3 (GraphPad Software, Boston, MA, USA).

## 3. Results

**Figure 1:**
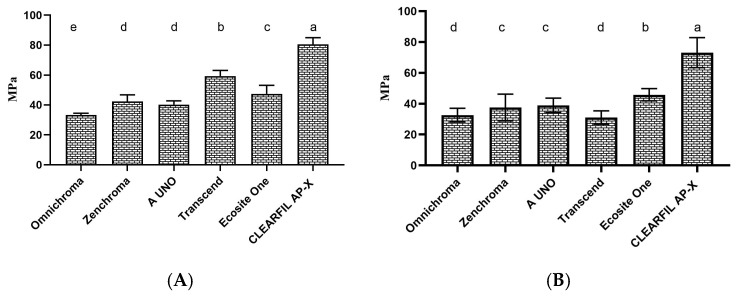
(**A**) Vicker’s hardness of the experimented materials after 24 h. (**B**) Vicker’s hardness of the experimented materials after 1 year. Groups denoted by identical letters did not exhibit statistically significant differences (*p* > 0.05).

The repeated measure ANOVA showed a statistically significant effect of aging on the HV of the experimental materials (*p* < 0.05). The types of composites and aging showed a significant interaction with an effect size η^2^p = 0.619. The MHRC showed a significantly higher HV than the USRC after 24 h (*p* < 0.05). Among the USRCs, TRANSCEND showed a significantly higher HV compared to other USRCs (*p* < 0.05). The mean HV values of all the experimented materials at 24 h are shown in Figure 1A. In case of 1-year aged specimen, the MHRC showed a significantly higher HV than the USRCs (*p* < 0.05). Among the USRC, Ecosite One showed a significantly higher HV compared to other USRCs (*p* < 0.05). The mean HV values of all the experimented materials at 1 year are shown in Figure 1B.

**Figure 2:**
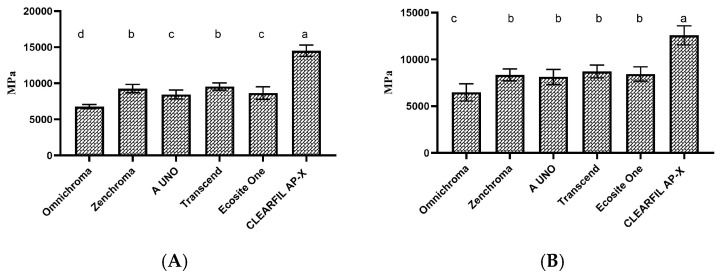
(**A**) Elastic modulus of the experimented materials after 24 h. (**B**) Elastic modulus of the experimented materials after 1 year. Groups denoted by identical letters did not exhibit statistically significant differences (*p* > 0.05).

The repeated measure ANOVA showed a statistically significant effect of aging on the EM of the experimental materials (*p* < 0.05). The types of composites and aging showed a significant interaction with an effect size η^2^p = 0.379. The MHRC showed a significantly higher EM than the USRCs after 24 h (*p* < 0.05). Among the USRCs, both the Transcend and Zenchroma showed a significantly higher HV compared to other USRCs (*p* < 0.05). Omnichroma showed the lowest EM among the tested materials. The mean EM values of all the experimented materials at 24 h are shown in Figure 2A. In case of 1-year aged specimen, the MHRC showed a significantly higher EM than the USRCs (*p* < 0.05). Among the USRCs, Omnichroma showed a significantly lower EM value compared to other USRCs (*p* < 0.05); however, there was no significant difference among other USRCs (*p* > 0.05). The mean EM values of all the experimented materials at 1 year are shown in Figure 2B.

**Figure 3:**
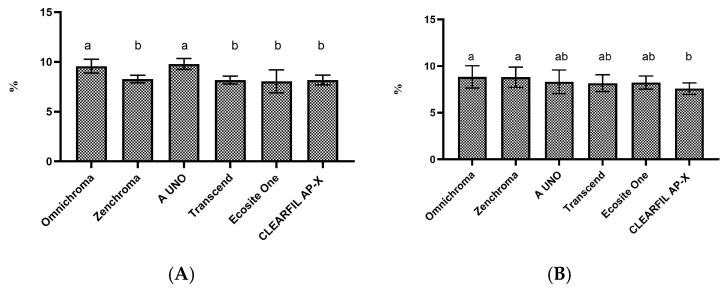
(**A**) Creep of the experimented materials after 24 h. (**B**) Creep of the experimented materials after 1 year. Groups denoted by identical letters did not exhibit statistically significant differences (*p* > 0.05).

The repeated measure ANOVA showed a statistically significant effect of aging on the CR% of the experimental materials (*p* < 0.05). The types of composites and aging showed a significant interaction with an effect size η^2^p = 0.450. Among the tested materials, Omnichroma and A UNO showed a significantly higher CR% compared to other materials (*p* < 0.05). The CR% of the MHRC showed an insignificant CR% compared to other USRCs (*p* > 0.05). The mean CR% values of all the experimented materials at 24 h are shown in Figure 3A. In case of 1-year aged specimen, the MHRC showed the least CR% among the tested materials. Omnichroma and Zenchroma showed the highest CR%. The mean CR% values of all the experimented materials at 1 year are shown in Figure 3B.

**Figure 4:**
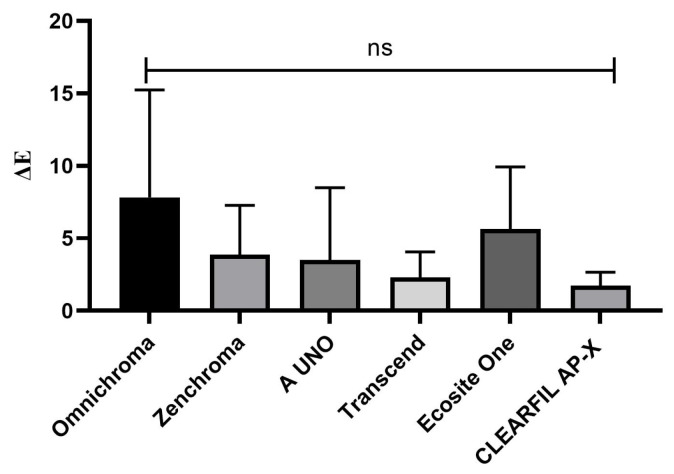
Color changes in the experimented materials after 1 year. Groups did not exhibit a statistically significant differences (*p* > 0.05).

The Kruskal–Wallis Test revealed that the color changes ΔE among the tested groups were statistically insignificant (*p* > 0.056). Among the tested materials, Omnichroma showed the highest and Clearfil APX showed the least color change. The ΔE of each experimental material is shown in Figure 4.

**Figure 5:**
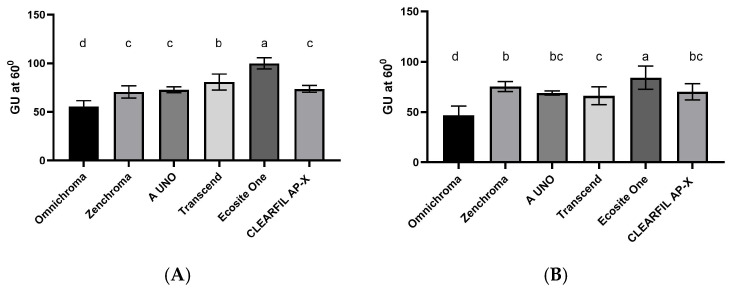
(**A**) Gloss value of the experimented materials after 24 h. (**B**) Gloss value of the experimented materials after 1 year. Groups denoted by identical letters did not exhibit statistically significant differences (*p* > 0.05).

The repeated measure ANOVA showed a statistically significant effect of aging on the GL of the experimental materials (*p* < 0.001). The types of composites and aging showed a significant interaction with an effect size η^2^p = 0.540 and. Among the tested materials, USRC Ecosite One showed the highest GL value and Omnichroma showed the lowest GL value at 24 h and 1-year aged specimens. The mean GL values of all the experimented materials at 24 h are shown in Figure 5A and those at 1 year are shown in Figure 5B.

**Figure 6:**
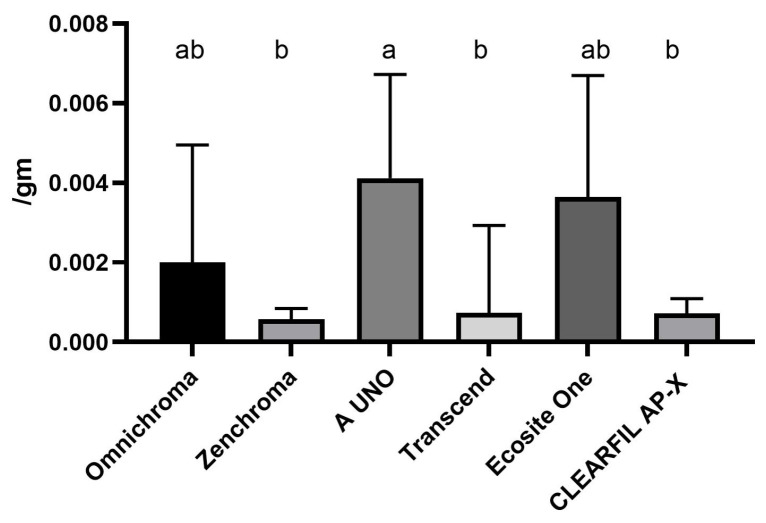
Solubility of the experimented materials after 1 year. Groups denoted by identical letters did not exhibit statistically significant differences (*p* > 0.05).

One-Way ANOVA showed a statistically significant effect of aging on the SL of the experimental materials (*p* < 0.02) and the effect size η^2^p = 0.319. The MHRC Clearfil APX, along with USRC Zenchroma and Transcend, showed the least SL after a 1-year period. USRC A-Uno showed the highest SL among the tested materials. The mean SL value of all the experimented materials is shown in Figure 6. The quantitative data of each experiment shown in Table 2. 

## 4. Discussion

This study comprehensively evaluated the mechanical, esthetic, and mass stability properties of five USRCs and one MHRC before and after simulated one-year aging via 10,000 thermocycles. The null hypothesis that aging would have no significant effect on the tested properties was rejected for most outcomes, which is consistent with well-documented evidence of the degradative effects of thermal cycling on resin-based materials.

In our study, the MHRC Clearfil APX demonstrated significantly higher HV and EM than all USRCs at both 24 h and 1-year evaluation intervals. This finding about MHRC aligns with the established mechanistic principle that composites with higher and more heterogeneously distributed filler loading exhibit superior hardness and stiffness [27]. González-Alenda et al. reported that conventional composites outperformed USRC in elastic modulus and hardness, attributing this to the dense, irregular filler packing of MHRC formulations compared to the uniformly sized spherical fillers in USRC [28]. Among the USRCs, Transcend showed the highest HV at baseline, which is consistent with its classification as a nano-hybrid formula with optimized filler architecture and higher degree of conversion. However, after aging, Ecosite One emerged as the hardest USRC, suggesting differential resistance of its filler–matrix interface to hydrolytic degradation during thermocycling [29]. Thermocycling induces mechanical degradation primarily through cyclic osmotic stress, plasticization of the resin matrix by water uptake, and hydrolysis of the silane coupling agent at the filler–matrix interface [30]. Omnichroma consistently showed the lowest EM both at baseline and after aging, which is mechanistically attributable to its unique architecture of uniformly sized (260 nm) spherical zirconia silica fillers that are specifically engineered to generate structural color through Bragg diffraction rather than optimize mechanical reinforcement [31]. This structural color mechanism inherently limits filler packing density and heterogeneity, compromising the percolation network required for maximum stiffness. Another study conducted by Yilmaz Atali et al. similarly reported that Omnichroma had the lowest elastic modulus among tested USRC, supporting the premise that its optical design is the principal trade-off against mechanical performance [32].

Omnichroma and A-UNO showed significantly higher creep recovery (CR%) at baseline, while Omnichroma and Zenchroma retained the highest CR% after aging. Higher CR% in USRC may reflect lower crosslink density and a more viscoelastic resin matrix, which confers resilience under cyclic loading but may compromise dimensional stability under sustained occlusal stress. The post-aging inversion, whereby Clearfil APX exhibited the lowest CR%, may reflect progressive matrix densification or continued crosslinking during extended aqueous storage, whereas USRC matrices undergo greater water-induced relaxation, increasing viscoelastic compliance [33]. Gunawan and Choi reported that multi-shade composites maintained higher hardness after 10,000 thermocycles, while single-shade composites showed comparatively greater susceptibility to property change, consistent with the CR% dynamics observed in the present study [34].

The Kruskal–Wallis analysis revealed no statistically significant differences in color change (ΔE) among tested groups after aging. All experimented materials demonstrated acceptable color stability under thermal aging in water. Omnichroma exhibited the highest ΔE, whereas Clearfil APX showed the least change. This finding partially contrasts with reports that position Omnichroma as highly color stable. One study found Omnichroma to have the lowest color change value among all tested composites after immersion in staining solutions [11]. This discrepancy may be explained by the different nature of the aging challenges. The present study employed thermocycling in distilled water, which stresses hydrolytic rather than extrinsic pigment-uptake pathways, whereas the referenced studies used staining media such as tea and red wine. The higher ΔE of Omnichroma under thermocycling in the present investigation may be related to its high translucency. Water permeates the filler–matrix interface during cycling, and the refractive index mismatch is altered, affecting light scattering and color perception [35]. Fidan et al. similarly noted that thermocycling significantly alters the color in USRC and was found to have greater color-changing potential than multi-shade counterparts [36]. Nevertheless, the overall non-significant intergroup differences observed here suggest that thermocycling in water alone does not produce clinically discriminatory color degradation across these material categories within one year of simulated service.

Thermocycling significantly reduced GL across all materials, with Ecosite One maintaining the highest and Omnichroma recording the lowest GL values at both time points. GL reduction is mechanistically driven by selective leaching of softer resin matrix components from the surface and differential filler protrusion, generating a filler-rich topography that increases light scattering and reduces specular reflectance. This mechanism has been confirmed in multiple thermocycling studies, with thermocycling consistently inducing gloss reduction that is material- and time-dependent [37]. Parallel evidence from the chemical degradation literature shows that initial gloss values are material-dependent and decline after aqueous aging, particularly in composites with less tightly packed filler networks [38]. The superior gloss retention of Ecosite One may reflect a finely tuned filler particle size distribution that resists preferential matrix dissolution and maintains surface smoothness. Conversely, the structural uniformity of Omnichroma’s spherical nanofillers, despite conferring excellent polish ability immediately after polishing, may create a surface more susceptible to localized matrix erosion during repeated thermal cycles, consistent with reports that Omnichroma’s surface roughness changes significantly after thermocycling [39]. The interaction between composite type and aging with a large effect size (η^2^p = 0.540) underscores that gloss degradation trajectories are highly material-specific, an important clinical consideration for posterior esthetic restorations.

Clearfil APX, Zenchroma, and Transcend demonstrated the least solubility after one year, whereas A-UNO exhibited the highest solubility. Higher solubility in certain USRCs is mechanistically linked to a greater initial concentration of unreacted monomers and oligomers that create osmotic gradients favoring water uptake and subsequent leaching, coupled with accelerated filler debonding when the silane layer hydrolyzed under cyclic thermal stress. Islam et al. reported that resin composites with different filler characteristics demonstrate significant differences in solubility, with filler content and interfacial bonding quality being primary determinants [16]. The comparable low solubility of Clearfil APX aligns with its established micro-hybrid filler architecture that provides dense interfacial contact and limits water diffusion pathways. Alkhudhayri et al. found that the water solubility and sorption of single-shade composites, including Omnichroma, were comparable to conventional nano-hybrid composites, though both showed increased surface roughness post-solubility testing [40]. The relatively higher solubility of A-UNO may reflect either a higher proportion of hydrophilic resin monomers or a less densely interconnected filler network that facilitates aqueous access to the organic matrix. Since excessive solubility leads to surface deformation, marginal degradation, and dimensional instability, the superior mass stability of Transcend and Clearfil APX is a clinically favorable attribute for stress-bearing restorations.

The differences in color stability and gloss retention observed among the tested materials may be partially attributed to variations in their resin matrix composition and filler characteristics. Resin composites containing more hydrophilic monomers, such as TEGDMA, are generally associated with increased water sorption and greater susceptibility to staining, whereas more hydrophobic monomers, such as UDMA and Bis-EMA, tend to exhibit improved color stability. Furthermore, filler loading, particle size, and filler morphology influence surface smoothness, wear resistance, and gloss retention [41].

Overall, while USRC offers compelling advantages in shade-matching efficiency and esthetic integration, their generally inferior mechanical performance relative to MHRC underscores the importance of material selection based on clinical context. From a clinical perspective, the results suggest that universal-shade resin composites are not interchangeable with respect to their long-term esthetic performance. Materials exhibiting superior color stability and gloss retention may be preferred for anterior restorations and other esthetically demanding situations where long-term appearance is critical. In contrast, materials showing greater susceptibility to discoloration or gloss loss may be more suitable for posterior restorations, small conservative restorations, or cases where periodic repolishing and maintenance can be readily performed. The large material-by-aging interaction effects observed across most outcome measures confirm that aging does not uniformly degrade all composites and that clinically meaningful material-specific differences emerge over time. Future research should focus on validating these findings through well-designed long-term clinical studies that assess the color stability, gloss retention, wear resistance, and patient-reported esthetic outcomes of universal-shade resin composites under intraoral conditions. From an in vitro perspective, more comprehensive aging models incorporating thermocycling, mechanical loading, toothbrushing simulation, staining challenges, and pH cycling may better replicate the complex oral environment. Limitations of the present investigation include its in vitro nature, the use of thermocycling as the sole aging modality, and specimen thickness, which may not fully replicate the complex multiaxial degradation occurring in the oral environment involving salivary proteins, dietary acids, mechanical wear, and bacterial challenge. Future studies incorporating combined aging protocols and long-term clinical trials are warranted to validate these findings.

## 5. Conclusions

Within the limitation of in vitro study model, the current research concludes that the USRC poses inferior mechanical properties compared with the convention MHRC. Thus, their use should be limited when strong and durable machinal properties are required under heavy occlusal stress. The mechanical and esthetic properties of USRC varied among different brands and these properties showed degradation over time. These differences appear to be influenced by material-specific characteristics, including resin matrix composition, filler loading, filler morphology, particle size distribution, and shade-matching technology. Ecosite One containing a radiopaque nanoparticle-based filler system emerged as the best-performing USRC mechanically and in terms of gloss retention over time. Omnichroma consistently exhibited the lowest mechanical properties and surface gloss, reflecting the inherent trade-off associated with its structural color mechanism. The USRC showed that a comparable color retention over time could be beneficial for achieving a highly esthetic outcome of the restoration that does not include the area that comes under heavy occlusal stress.

## Figures and Tables

**Table 1:** The composition and classification of the tested materials.

Material	Manufacturer	Type/Classification	LOT Number	Filler Load (wt%)
**Omnichroma**	Tokuyama Dental, Japan	Single-shade resin composite composed of a UDMA/TEGDMA resin matrix and 260 nm spherical silica-zirconia fillers	171E04	79
**Zenchroma**	President Dental, Germany	Micro hybrid universal-shade resin composite composed of a Bis-GMA/UDMA/TMDMA resin matrix and inorganic glass powder and silicon dioxide fillers	2121009698	75
**A-Uno**	Yamakin, Japan	Universal-shade, fluoride-releasing resin composite containing a methacrylate-based resin matrix and silica/glass fillers	30112302	74
**TRANSCEND**	Ultradent, South Jordan, UT, USA	Universal-shade resin composite formulated with a proprietary blend of resin monomers and inorganic filler particles	BW93R	79
**Ecosite One**	DMG, Germany	Nano-hybrid universal-shade resin composite incorporating a radiopaque nanoparticle-based filler system and proprietary NC1 (Non-Clustering) technology	310802	82
**Clearfil AP-X** **(A2)**	Kuraray Noritake, Japan	Micro-hybrid resin composite composed of a Bis-GMA/TEGDMA resin matrix and silanated barium glass, silica, and colloidal silica fillers	BN0042	86

**Table 2:** Mechanical and esthetic outcome of each material. Mean, standard deviation and statistical significance of each experiment. Groups denoted by identical letters did not exhibit statistically significant differences (*p* > 0.05).

Test		Material					
		Omnichroma	Zenchroma	A-Uno	TRANSCEND	Ecosite One	Clearfil AP-X
HV MPa	24 h	33.2 ± 1.2 e	42.3 ± 4.4 d	40.1 ± 2.5 d	59.3 ± 3.7 b	47.3 ± 5.8 c	80.4 ± 4.5 a
	1 year	32.6 ± 4.4 d	37.5 ± 8.7 c	38.9 ± 4.6 c	31.1 ± 4.3 d	45.8 ± 3.9 b	73.1 ± 9.8 a
EM MPa	24 h	6768 ± 305 d	9275 ± 566 b	8465 ± 607 c	9558 ± 501 b	8645 ± 882 c	14520 ± 778 a
	1 year	6477 ± 915 c	8342 ± 641 b	8125 ± 810 b	8707 ± 689 b	8443 ± 756 b	12578 ± 1017 a
CR %	24 h	9.57 ± 0.69 a	8.26 ± 0.38 b	9.78 ± 0.55 a	8.16 ± 0.39 b	8.03 ± 1.14 b	8.17 ± 0.48 b
	1 year	8.83 ± 1.19 a	8.80 ± 1.07 a	8.31 ± 1.27 ab	5.86 ± 0.89 ab	8.23 ± 0.70 ab	7.57 ± 0.60 b
∆E		7.81 ± 7.41 ns	3.87 ± 3.39 ns	3.49 ± 4.99 ns	2.29 ± 1.76 ns	5.63 ± 4.28 ns	1.72 ± 0.93 ns
GL	24 h	55.70 ± 5.83 d	70.58 ± 6.26 c	72.79 ± 3.08 c	80.78 ± 8.17 b	99.94 ± 5.73 a	73.66 ± 3.48 c
	1 year	46.88 ± 9.13 d	75.31 ± 4.99 b	68.84 ± 2.08 bc	66.21 ± 8.78 c	84.16 ±11.65 a	70.21 ± 7.99 bc
SL		0.00199 ± 0.0029 ab	0.00057 ± 0.0002 b	0.00411 ± 0.0026 a	0.00073 ± 0.0021 b	0.00364 ± 0.0030 ab	0.00072 ± 0.0003 b

## Data Availability

The original contributions presented in this study are included in the article. Further inquiries can be directed to the corresponding author.

## Abbreviations

The following abbreviations are used in this manuscript:

USRC	Universal-shade resin composites
MHRC	Micro-hybrid resin composite
RC	Resin composite
HV	Vicker’s micro-hardness
EM	Elastic modulus
CR	Creep
GL	Gloss
SL	Solubility
